# Influence of cardiovascular risk-factors on morphological changes of cerebral arteries in healthy adults across the life span

**DOI:** 10.1038/s41598-021-91669-3

**Published:** 2021-06-10

**Authors:** Pauline Mouches, Sönke Langner, Martin Domin, Michael D. Hill, Nils D. Forkert

**Affiliations:** 1 Department of Radiology, Faculty of Medicine, University of Calgary, Calgary, Canada; 2 Institute for Diagnostic Radiology and Neuroradiology, University Medical Center Rostock, Rostock, Germany; 3 Functional Imaging Unit, Institute for Diagnostic Radiology and Neuroradiology, University Medicine Greifswald, Greifswald, Germany; 4 Department of Clinical Neurosciences, University of Calgary, Calgary, Canada; 5 Hotchkiss Brain Institute, University of Calgary, Calgary, Canada; 6 Alberta Children’s Hospital Research Institute, University of Calgary, Calgary, Canada; 7 Department of Community Health Sciences, University of Calgary, Calgary, Canada

**Keywords:** Cerebrovascular disorders, Anatomy, Risk factors, Brain imaging

## Abstract

Cerebral artery morphological alterations have been associated with several cerebrovascular and neurological diseases, whereas these structures are known to be highly variable among healthy individuals. To date, the knowledge about the influence of cardiovascular risk factors on the morphology of cerebral arteries is rather limited. The aim of this work was to investigate the impact of cardiovascular risk factors on the regional cerebroarterial radius and density. Time-of-Flight magnetic resonance angiography from 1722 healthy adults (21–82 years) were used to extract region-specific measurements describing the main cerebral artery morphology. Multivariate statistical analysis was conducted to quantify the impact of cardiovascular risk factors, including clinical and life behavioural factors, on each region-specific artery measurement. Increased age, blood pressure, and markers of obesity were significantly associated with decreased artery radius and density in most regions, with aging having the greatest impact. Additionally, females showed significantly higher artery density while males showed higher artery radius. Smoking and alcohol consumption did not show any significant association with the artery morphology. The results of this study improve the understanding of the impact of aging, clinical factors, and life behavioural factors on cerebrovascular morphology and can help to identify potential risk factors for cerebrovascular and neurological diseases.

## Introduction

Aging is one of the most important risk factors for cerebrovascular diseases, such as acute ischemic strokes^1^. Due to the aging population, a significant increase of cerebrovascular diseases can be expected in the future. In addition to aging, several other cardiovascular risk factors have been associated with an increased risk for cerebrovascular alterations in the past, including a high body mass index (BMI) or elevated blood pressure, suggesting that these cardiovascular risk factors are also associated with cerebrovascular diseases^2,3^. For instance, patients with atherosclerosis, which leads to a thinner inner lumen of arteries, have been reported to have a higher risk for dementia and stroke^4,5^. Hence, a detailed understanding of the effects of cardiovascular risk factors on cerebrovascular structures is necessary to improve the prevention of cerebrovascular diseases.

Investigating the effects of cardiovascular risk factors on cerebrovascular structures, especially arteries, is a challenging task because not only are cardiovascular risk factors often highly correlated but the healthy cerebrovascular system is also highly variable among individuals, which complicates a detailed morphological analysis^6,7^. For this reason, previous studies mostly focused on molecular, cellular, and functional changes of the cerebrovascular system associated with aging and other cardiovascular risk factors^8^. Within this context, changes in artery wall composition and stiffness, and cerebral blood flow have been found to be associated with aging and other cardiovascular risk factors^9,10^. However, artery morphology is directly related to blood flow with lower radius (r) being associated with higher resistance (R ≈ 1/r^4^) and, thus, decreased blood flow (BF ≈ 1/R).

Only a few studies have investigated the artery morphology using angiography datasets so far. Bullitt et al.^11^, for example, investigated the effects of aging and sex on cerebrovascular structures in four different brain regions by computing and comparing the number of vessels, average radius, and tortuosity using magnetic resonance angiography (MRA) datasets of 100 healthy volunteers aged 18–74. They reported a decreasing vessel density with aging as well as an increased vessel radius in males compared to females. However, potentially due to the rather small sample size, they used an age-group based statistical approach instead of regression and did not consider the total brain volume in their analysis of artery radii, which might bias the results. More recently, Williamson et al.^3^ studied the impact of various cardiovascular risk factors on brain vessel density and caliber using MRA datasets of 125 young adults (aged 18–40 years). They reported a negative correlation of body-mass-index and systolic blood pressure with vessel density, and a positive correlation of smoking with vessel density. Additionally, they found the vessel caliber to be negatively correlated with systolic blood pressure and a positive correlation with smoking. Nevertheless, they studied the whole brain without analyzing regional effects while the study cohort was also limited with respect to the age range.

This study aims to quantify and analyze the regional artery morphology in a large cohort of healthy volunteers and identify associations between the artery morphology and age, sex, and other cardiovascular risk factors using a multivariate approach. Additionally, differences in regional artery morphology of subjects with and without fetal posterior cerebral artery (PCA)^12^ were investigated.

## Materials and methods

### Data

All datasets included in this study were collected in the Study of Health in Pomerania (SHIP)^13^. The aim of SHIP was to obtain a representative general population sample to assess incidence and prevalence of common risk factors as well as of subclinical and clinical disease to investigate the complex relationships between these factors and conditions. The participants were selected from the counties of Nord- and Ostvorpommern and the two cities of Greifswald and Stralsund in Northeast Germany. Inclusion criteria were primary place of residence within the target area and age of 20–79 at the time of sampling. Out of 6265 eligible individuals of the first cohort, 4308 participated (68.8%) in the baseline examination (SHIP-0, 1997–2001). Follow-up examinations took place between 2002 and 2006 (SHIP-1, N = 3300) and between 2008 and 2012 (SHIP-2, N = 2333). An independent cohort, SHIP-Trend, was established in 2008. A stratified sample of 10,000 individuals was drawn from the central population registry, 8826 were eligible, and 4420 participated (response rate of 50.1%)^14^. SHIP-2 and SHIP-Trend participants formed the basis for the current analysis. The database used for this study consists of 2115 healthy adults, aged 21 to 82 years. All MRA datasets were acquired on a 1.5T system (Magnetom Avanto; Siemens Medical Solutions, Erlangen, Germany) using a Time-of-Flight (TOF) MRA sequence with the following acquisition parameters: TR = 23 ms, TE = 7 ms, flip angle = 25°, spacing = 0.7 × 0.7 × 0.7 mm^3^, scan time = 3:23 min. Moreover, T1-weighted MRI datasets of each participant were also acquired on the same scanner with the following acquisition parameters: TR = 1900 ms, TE = 3.4 ms, flip angle = 15°, spacing = 1.0 × 1.0 × 1.0 mm^3^, scan time = 3:38 min. The TOF MRA and T1-weighted MRI datasets were acquired as part of a whole-body MRI examination, with a mean scan time of 90 min per participant^15^. All participants underwent thorough medical questionnaires and examinations^14^.

In addition to imaging data, clinical and behavioural parameters were collected at the time of examination for each participant. The available clinical factors for this study include age, sex, body-mass-index (BMI; kg/m^2^), waist-to-hip ratio (WHR), systolic blood pressure (mmHg) and heart rate (beats per minutes [BPM]) at rest, both averaged over three measurements. The behavioural factors used in this study include smoking history (years of smoking) and alcohol consumption (average number of drinks per day).

All study protocols and procedures were conducted in accordance to the ethical guidelines and in compliance with the Declaration of Helsinki. Each participant gave written informed consent and the local ethics commission of the University of Greifswald approved the original SHIP study (BB 39/08, 19.06.2008).

### Data preprocessing

For quantitative analysis of the cerebroarterial system, the vessels were automatically segmented in each TOF MRA dataset using a level-set based segmentation method described by Forkert et al.^16^.

**Figure 1:**
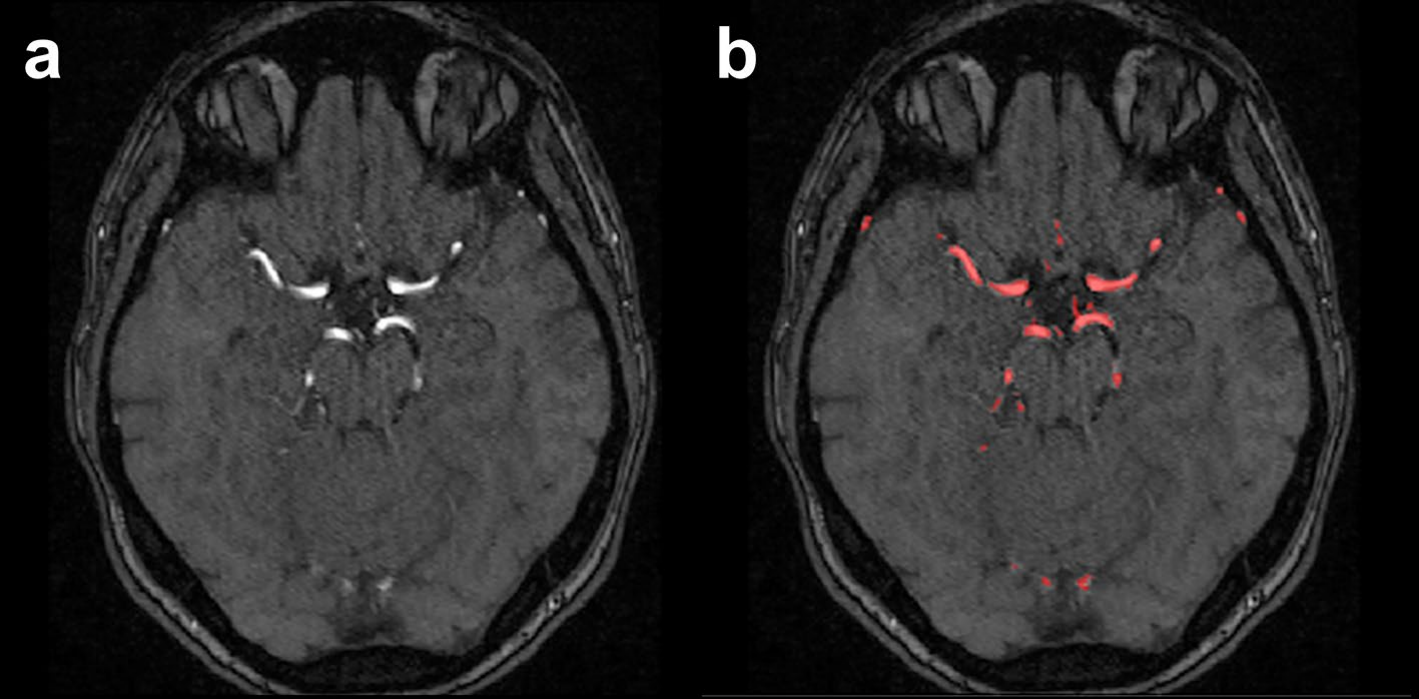
Vessel segmentation overlaid onto a TOF MRA dataset. (**a**) Example TOF MRA dataset. (**b**) Corresponding vessel segmentation in red.

Next, the MNI brain atlas^17^ was registered to each subject-specific T1-weighted MRI dataset using a non-linear transformation. After this, each subject-specific T1-weighted MRI dataset was registered to its corresponding TOF MRA dataset using a linear transformation. These registrations were performed using the Advanced Normalization Tools (ANTs) toolkit^18^. Finally, these two transformations were concatenated, which allows transforming atlas information and brain regions defined in the MNI atlas directly to each subject-specific TOF MRA dataset. This concatenated transformation was used to warp the probabilistic cerebrovascular atlas described by Mouches and Forkert^6^ to each individual TOF MRA dataset, which was then used to refine the automatic vessel segmentation. More precisely, any segmented vessel voxel exhibiting an occurrence probability below 1% in the probabilistic cerebrovascular atlas was removed, which allows cleaning noise-related over-segmentations from the vessel segmentations. Figure 1 shows an example of segmented vessels.

In the next step, the 3D centerlines of the segmented vessels were extracted using the method described by Lee et al.^19^. The distance between each centerline voxel and its closest vessel boundary voxel was computed based on the distance transform described by Danielson^20^. This distance was then used to define the vessel radius for each centerline voxel.

Finally, the total intracranial volume of each participant was determined. Therefore, the intracranial segmentation mask defined in the MNI atlas was transformed to each participant T1-weighted dataset using the transformation determined during the registration process. The intracranial volume of each dataset was measured by the number of voxels in the mask multiplied by the spatial resolution. This metric was used as the only imaging factor together with the clinical and behavioural parameters to evaluate its influence on the artery morphology.

### Regional artery analysis

**Figure 2:**
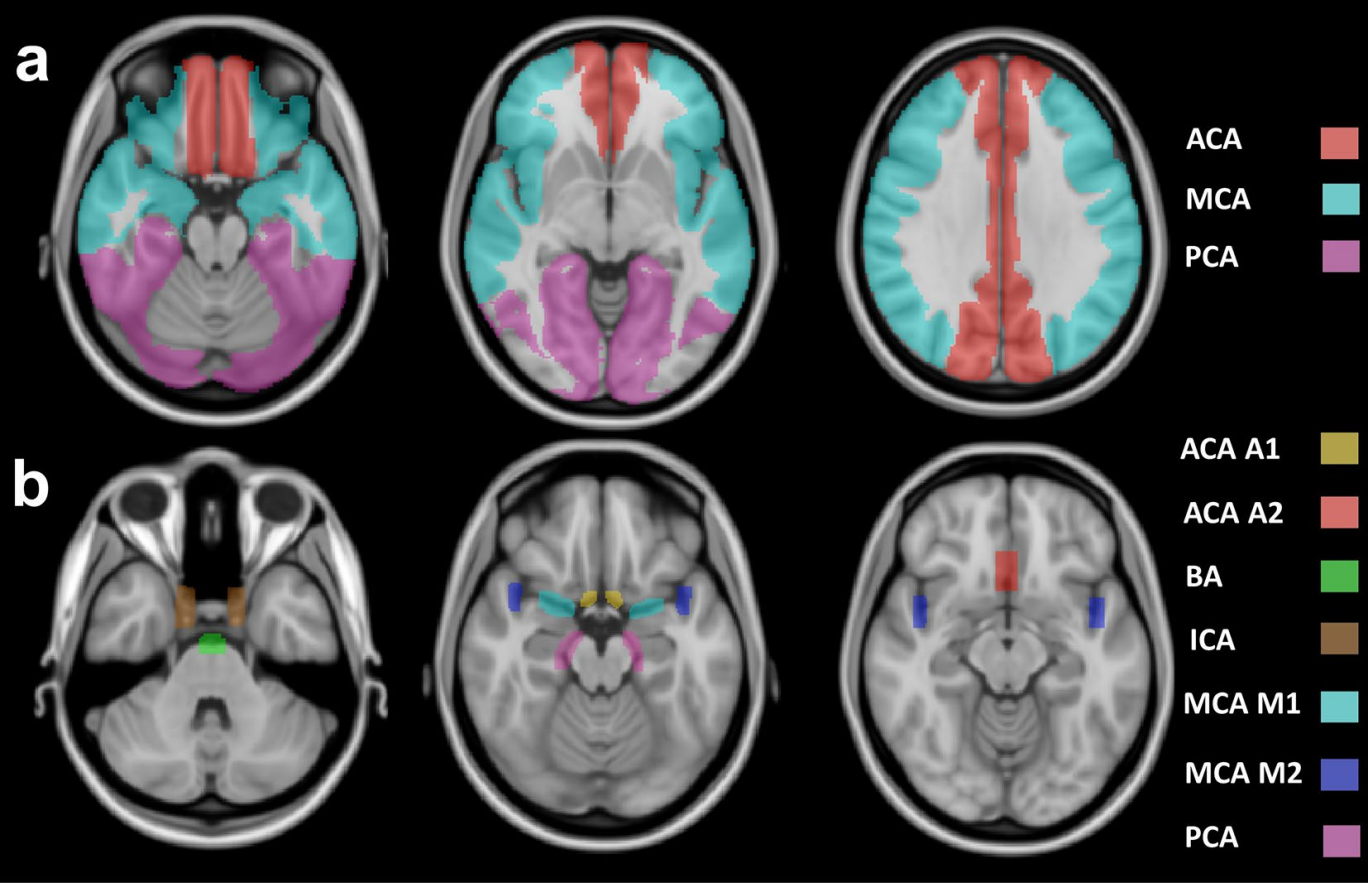
Regions-of-interest for artery morphology measurements overlaid onto the MNI brain atlas. (**a**) Flow territories for artery density and mean artery radius measurements. (**b**) Main artery masks for mean radius measurements. ACA: anterior cerebral artery, PCA: posterior cerebral artery, MCA: middle cerebral artery, ICA: internal carotid artery, BA: basilar artery.

Two different types of regional artery morphology measurements were used in this study (Fig. 2). In the first case, the artery density and average artery radius were measured in each of the three main blood flow territories in the brain: the anterior cerebral artery (ACA) territory, the posterior cerebral artery (PCA) territory, and the middle cerebral artery (MCA) territory, separately for the left and right hemisphere. To do so, a flow territory atlas^21^ defined in MNI space was warped to each participant TOF MRA dataset. The proximal, middle, and distal part of each main artery territory defined in the original atlas were merged to single flow territory masks to reduce the effect of noise. For the computation of the artery density within a flow territory, the ratio of the volume of the segmented vessel voxels and volume of the corresponding flow territory was calculated. For calculation of the average artery radius within a flow territory, only the radius values of the centerline voxels were considered to prevent a biased average calculation due to large arteries.

For the second measurement type, small masks covering characteristic locations of the main arteries were manually defined in the probabilistic cerebrovascular atlas^6^ and transformed to each participant TOF MRA dataset. The arteries defined included: the anterior cerebral arteries (ACA) (A1 and A2 segments), the posterior cerebral arteries P2 segment (PCA), the middle cerebral arteries (MCA) (M1 and M2 segments), the basilar artery (BA), and the internal carotid arteries cavernous segment (ICA). All arteries except for the BA and ACA A2 segment were defined in the left and right hemisphere. For quantitative evaluation, the mean artery radius was extracted for each artery mask using the corresponding radius values at the centerline voxels. No artery density was calculated for this second measurement type as the masks were restricted to small regions encompassing only the major arteries.

### Quality control

The vessel segmentations of each dataset were visually assessed using a three-dimensional rendering of the segmented vessel. Additionally, the vessel anatomy was visually inspected to identify participants with fetal PCA. Finally, the registration of the MNI brain atlas to each TOF MRA dataset were visually evaluated by overlaying the registered main artery masks onto the vessel segmentations for each participant.

### Statistical analysis

Multivariate analysis of covariance (MANCOVA) was used for group comparison of the participants with and without fetal PCA using the artery measurements as dependent variables, age and sex as covariates, and the class (fetal PCA yes/no) as the fixed factor. Although measurements from left and right hemispheres were included as dependent variables, none of the dependent variables showed very high correlation (r > 0.8). Pairwise comparisons of the dependent variables with Bonferroni correction (n = 24) were performed post-hoc to identify significant parameters. A p-value < 0.05 (i.e. Bonferroni-corrected p-value < 0.002) was considered significant.

Quantile local piecewise regression analyses (LOESS)^22^ were used to generate sex-specific plots with the age-related 25th, 50th, and 75th quantile curves individually for each region of interest and artery measurement.

After this, multivariate partial least squares regression (PLSR) was used to assess the ability of the non-imaging factors and total intracranial volume to predict the artery measurements extracted from the TOF MRA datasets. PLSR is a statistical approach that can be classified as a supervised dimensionality reduction method, in which the dimensionality reduction process aims to maximize the correlation with the response variable^23^. More precisely, this approach identifies a set of uncorrelated latent components that compile the predictor variables with a maximal variation and that simultaneously have a maximum correlation with the response variable. In case of multivariate PLSR, several response variables can be used. PLSR is a suitable statistical analysis in this study as it can handle collinear predictor variables by generating and analyzing uncorrelated latent components instead of the predictor variables themselves.

A leave-one-out cross validation process was used to ensure the internal validity of the results. Briefly described, as many models as number of participants were trained using this approach. Each model was trained on the entire population except for one participant who was used for testing the model. The process resulted in several models, which were combined by averaging of the testing errors. The averaged error was used to determine the optimal number of components to use in the PLSR model.

This statistical analysis was conducted using the pls package version 2.7-2 from R Project for Statistical Computing^24^ using the kernel algorithm method. The response variables were normalized between 0 and 1, and the predictor variables were centered and scaled before training the models. The root mean squared error (RMSE) was used to assess the models and select the optimal number of components. The importance of each predictor variable was assessed by evaluating its beta coefficient and associated p-value, which was computed using the jackknife sampling method ^25^, implemented within R.

In this study, three multivariate PLSR models were trained with different response variables: one with the artery density within each flow territory, one with the mean artery radius within each flow territory, and one with the mean radius of the main arteries.

## Results

### TOF dataset quality control

The quality control of the vessel segmentation performed on the TOF datasets resulted in the removal of 122 datasets. More precisely, 36 datasets were removed due to noisy segmentations, while 86 datasets were excluded because of incomplete segmentation. The quality control of the registrations between the TOF atlas and each TOF dataset resulted in the removal of 271 additional datasets, with most of the mis-registrations located around the ACA A1 segment. Thus, a total of 1722 datasets were finally included in this study, including 216 (12.5%) participants with fetal PCA in at least one hemisphere, and 1506 (87.5%) participants with a normal vessel anatomy. Table 1 reports the mean and standard deviation of the clinical characteristics of the participants finally included in this study. These characteristics are cross-sectional. They were collected on the day of the examination and do not take into account the history of the participants.

**Table 1:** Non-imaging factors.

	Mean (std)
**Clinical factors**	
Age	50.23 (13.83)
Sex (F: female; M: male)	F: 937 (54.4%); M: 785 (45.6%)
Heart rate at rest (BPM)	70.61 (10.40)
Systolic blood pressure at rest (mmHg)	126.52 (17.09)
Body-mass index (BMI; kg/m^2^)	27.48 (4.39)
Waist-to-hip ratio (WHR)	0.88 (0.09)
Intracranial volume (ICV; cm^3^)	1402.44 (136.10)
**Behavioral factors**	
Smoking duration (years)	9.55 (13.42)
Number of drinks per day (0: zero; 1: one to two; 2: three to four; 3: five to six; 4: seven to nine; 5: ten or more)	0: 123 (7.1%); 1: 1222 (71%); 2: 313 (18.2%); 3: 49 (2.8%); 4: 12 (0.7%); 5: 3 (0.2%)

Clinical and behavioral factor mean and standard deviation of the finally included subjects.

### Artery measurement comparison between participants with and without fetal PCA

Table 2 contains the age- and sex-adjusted mean and standard deviation of the extracted artery measurements for the participants with a normal cerebrovascular structure anatomy and those with fetal PCA. The MANCOVA analysis revealed a statistically significant difference between participants with and without a fetal PCA (p < 0.001). Pairwise comparisons revealed Bonferroni-corrected significant differences between the groups for the BA radius (p < 0.0001) and the right ICA radius (p < 0.0001). No other artery measurement was found to differ significantly (Bonferroni-corrected) between the participants with and without a fetal PCA (p > 0.002). More precisely, participants with a fetal PCA exhibit a smaller mean radius of the BA and larger mean radius of the right ICA. Based on these observations, the participants with fetal PCA were not considered for the subsequent analyses to prevent biased results due to this anatomical variation.

**Table 2:** Morphological measurements of the cerebral arteries.

	Normal anatomy group mean (std)		Fetal PCA variation group mean (std)	
	Left	Right	Left	Right
**Artery density in flow territories (%)**				
ACA	0.756 (0.006)	1.177 (0.008)	0.765 (0.017)	1.230 (0.021)
MCA	0.707 (0.004)	0.852 (0.005)	0.686 (0.004)	0.859 (0.012)
PCA	0.274 (0.003)	0.308 (0.003)	0.278 (0.007)	0.311 (0.008)
**Mean artery radius in flow territories (mm)**				
ACA	0.761 (0.003)	0.715 (0.002)	0.769 (0.007)	0.728 (0.005)
MCA	0.688 (0.001)	0.751 (0.002)	0.689 (0.003)	0.761 (0.004)
PCA	0.618 (0.002)	0.634 (0.002)	0.621 (0.004)	0.639 (0.004)
**Mean artery radius (mm)**				
ACA A1	1.025 (0.004)	0.992 (0.004)	0.997 (0.011)	0.991 (0.011)
ACA A2	0.890 (0.002)		0.879 (0.006)	
ICA	1.849 (0.007)	1.848 (0.007)	1.900 (0.018)	1.936 (0.019)*
MCA M1	1.193 (0.003)	1.168 (0.003)	1.196 (0.009)	1.174 (0.009)
MCA M2	0.811 (0.003)	0.787 (0.002)	0.808 (0.007)	0.787 (0.006)
PCA	0.906 (0.002)	0.898 (0.002)	0.886 (0.007)	0.891 (0.006)
BA	1.049 (0.004)		0.962 (0.010)*	

Morphological measurements of the cerebral arteries for the subjects with (12.5%) and without (87.5%) fetal PCA, adjusted for age and sex.

ACA: anterior cerebral artery, MCA: middle cerebral artery, PCA: posterior cerebral artery, ICA: Internal carotid artery, BA: Basilar artery.

*Significant difference between the two groups, after Bonferroni correction, p-value < 0.002.

### Effect of cardiovascular risk-factors on artery morphology

Age- and sex-specific regression plots for the participants without a fetal PCA are shown in the supplemental material (Supplemental Figures 1–3). Qualitatively, the plots suggest that a high variation between the participants remains for the different artery measurements even after accounting for age and sex.

Tables 3, 4, and 5 display the beta coefficients and their significance for the final PLSR models. The reported p-values were not adjusted for multiple comparisons. The optimal number of PLS components used for building the models was determined individually for each model by evaluating and comparing the cross-validated RMSE while iteratively adding more components to the model. Based on this analysis, three PLS components were used for each model. The RMSE values of the finally built models are shown in Tables 3, 4 and 5, for each predictor variable. Comparing the predictions for left and right hemisphere measurements revealed high RMSE differences in some cases, especially for the MCA flow territory artery density and for the mean ACA flow territory artery radius. However, there were no considerable differences in the beta coefficients magnitude, direction, and significance between the left and right hemisphere measurements.

**Table 3:** Partial least squares model—flow territory artery density.

	ACA		PCA		MCA	
	L	R	L	R	L	R
RMSE	0.1218	0.1168	0.1478	0.1292	0.1623	0.1234
ICV	− 0.004	**− 0.008**	− 0.004	− 0.004	− 0.006	0.002
Sex (M = 1; F = 2)	0.003	**0.007**	**0.009**	**0.008**	**0.016**	**0.008**
Age	**− 0.025**	**− 0.027**	**− 0.038**	**− 0.029**	− 0.005	0.000
BMI	0.000	− 0.001	**− 0.011**	**− 0.009**	**− 0.018**	**− 0.016**
WHR	− 0.002	*− 0.005*	**− 0.013**	**− 0.012**	**− 0.022**	**− 0.016**
Systolic blood pressure	*− 0.006*	**− 0.009**	**− 0.016**	**− 0.013**	**− 0.015**	**− 0.010**
Heart rate	0.002	0.002	*0.005*	*0.004*	0.003	0.003
Drinks per day	0.000	− 0.002	0.003	0.002	− 0.001	0.003
Smoking duration	0.002	0.002	− 0.001	− 0.001	− 0.004	− 0.005

Beta coefficients of the final partial least squares regression model predicting the artery density in the blood flow territories. Italic: p-value < 0.05, Bold: p-value < 0.01. ACA: anterior cerebral artery, PCA: posterior cerebral artery, MCA: middle cerebral artery, L: left, R: right.

**Table 4:** Partial least squares model—flow territory mean artery radius.

	ACA		PCA		MCA	
	L	R	L	R	L	R
RMSE	0.1433	0.08569	0.1389	0.1407	0.1095	0.1117
ICV	*0.006*	**0.008**	0.005	*0.006*	**0.020**	**0.028**
Sex (M = 1; F = 2)	**− 0.005**	*− 0.003*	− 0.001	− 0.001	**− 0.007**	**− 0.012**
Age	− 0.002	− 0.002	**− 0.017**	**− 0.016**	− 0.002	− 0.003
BMI	− 0.002	**− 0.006**	**− 0.007**	**− 0.008**	**− 0.013**	**− 0.018**
WHR	0.002	**− 0.002**	**− 0.006**	**− 0.006**	− 0.003	− 0.003
Systolic blood pressure	0.000	**− 0.004**	*− 0.005*	*− 0.005*	**− 0.009**	**− 0.011**
Heart rate	− 0.001	− 0.002	*0.006*	0.005	**− 0.006**	**− 0.008**
Drinks per day	0.002	− 0.001	*0.005*	0.004	− 0.004	− 0.005
Smoking duration	0.001	− 0.001	− 0.001	− 0.002	− 0.001	0.000

Beta coefficients of the final partial least squares regression model predicting the mean artery radius in the blood flow territories. Italic: p-value < 0.05; Bold: p-value < 0.01. ACA: anterior cerebral artery, PCA: posterior cerebral artery, MCA: middle cerebral artery, L: Left, R: Right.

**Table 5:** Partial least squares model—mean artery radius.

	ACA A1		ACA A2	PCA		MCA M1		MCA M2		ICA		BA
	L	R		L	R	L	R	L	R	L	R	
RMSE	0.1299	0.1491	0.1230	0.1176	0.1201	0.1168	0.1279	0.1075	0.1169	0.1135	0.1234	0.1036
ICV	**0.012**	**0.009**	0.004	**0.009**	**0.008**	0.007	0.010	**0.011**	**0.013**	**0.020**	**0.024**	**0.012**
Sex (M = 1; F = 2)	*− 0.004*	− 0.002	**0.006**	− 0.001	− 0.002	**− 0.008**	**− 0.009**	− 0.001	*− 0.003*	**− 0.010**	**− 0.011**	0.000
Age	− 0.005	*− 0.012*	**− 0.022**	**− 0.012**	**− 0.012**	**0.017**	**0.018**	**− 0.016**	**− 0.009**	0.000	− 0.005	**− 0.014**
BMI	**− 0.007**	− 0.005	**− 0.010**	**− 0.007**	− 0.003	− 0.001	− 0.004	**− 0.008**	**− 0.009**	**− 0.010**	**− 0.011**	**− 0.011**
WHR	*− 0.004*	− 0.003	**− 0.012**	**− 0.005**	− 0.002	*0.004*	0.003	**− 0.006**	**− 0.005**	− 0.002	− 0.003	**− 0.009**
Systolic blood pressure	*− 0.007*	− 0.004	**− 0.010**	**− 0.006**	− 0.002	− 0.001	− 0.003	**− 0.007**	**− 0.008**	**− 0.008**	**− 0.009**	**− 0.010**
Heart rate	− 0.002	0.001	0.004	0.001	0.002	**− 0.007**	**− 0.009**	0.001	− 0.002	**− 0.006**	*− 0.006*	0.000
Drinks per day	− 0.002	0.001	0.002	0.000	0.002	− 0.005	− 0.007	0.001	− 0.001	− 0.004	− 0.004	− 0.001
Smoking duration	− 0.002	− 0.001	0.000	− 0.001	0.000	− 0.002	− 0.002	− 0.001	− 0.002	− 0.003	− 0.003	− 0.002

Beta coefficients of the final partial least squares regression model predicting the artery radius of the major cerebral arteries. Italic: p-value < 0.05; Bold: p-value < 0.01. ACA: anterior cerebral artery, PCA: posterior cerebral artery, MCA: middle cerebral artery, ICA: internal carotid artery, BA: Basilar artery, L: left, R: right.

For the artery density (Table 3), all three flow territories showed lower density associated with higher blood pressure, and greater artery density in females than males (p < 0.01, except in left ACA). While increased age was associated with a reduced artery density in the ACA and PCA territories (p < 0.01), the MCA territory artery density was not significantly affected by age. The artery density in the MCA and PCA territories was found to be lower with increased BMI and WHR (p < 0.01). Higher heart rate was associated with higher density in the PCA flow territory (p < 0.05). Smoking duration and alcohol consumption did not show any significant association. Higher intracranial volume was mostly non-significantly associated with lower artery density measurements.

The mean artery radius in the flow territories (Table 4) showed similar results as the artery density for the BMI and WHR factors with a lower artery radius being associated with higher BMI and WHR in most territories. Increased age was associated with lower mean artery radius in all flow territories, but only significantly in the PCA flow territory (p < 0.01). In contrast to the artery density, which overall was larger in females, the mean artery radius was generally larger in males. Higher systolic blood pressure was also associated with a reduced artery mean radius, as well as heart rate in the MCA flow territory (p < 0.01). Finally, alcohol consumption was associated with greater mean artery radius in the left PCA flow territory (p < 0.05) while smoking did not show any significant association. In contrast to the artery density, higher intracranial volume was associated with higher mean artery radius in the flow territories.

For the main artery radius measurements (Table 5), age and total intracranial volume showed the greatest impact in general, with greater intracranial volume being associated with increased artery radii while increased aging was associated with lower artery radii (p < 0.01), except for the MCA M1 segment and the ICA. Overall, increased BMI and WHR were associated with a smaller radius in most arteries, especially in the ACA A2, MCA M2, and BA (p < 0.01). Increased systolic blood pressure and heart rate were also mostly associated with smaller artery radii. Smoking and alcohol effects were not significant in any of the regions. Sex showed significant effects in the ACA A2, ICA, and MCA M1 (p < 0.01), with increased radii in females in the ACA A2, and in the ICA and MCA M1 for males.

## Discussion

This is the first study that investigates the association of cardiovascular risk factors and cerebral artery morphology using a large database of healthy volunteers across the whole adult age spectrum using automatic and robust image analysis methods. Evaluating the independent effects of highly correlated variables is a challenging task that requires the use of advanced statistical methods. Within this context, the use of a PLSR approach in this field is novel. This statistical approach was successfully used in the past in various other domains, for example in neuroimaging to analyze the associations between brain activity and behaviour^26^.

### Effect of fetal PCA variation on artery radius and density

The results of this study suggest that there is a significant difference in the mean artery radius between participants with and without fetal PCA, whereas subjects with fetal PCA have generally smaller BA radius and a larger right ICA radius. Other arteries, such as the PCA and ACA, were also found to be smaller in subjects with fetal PCA although these results were not significant. In patients with a fetal PCA, the PCA is mainly connected to the ICA instead of the BA in healthy subjects without a fetal PCA, which explains the radius differences found. These findings are also in line with previous studies investigating blood flow in subjects with fetal PCA, which showed reduced blood flow in the BA and increased blood flow in the ICA^10^. Furthermore, subjects with fetal PCA generally showed higher vessel density and mean radius in all flow territories although these differences were not significant and, to our knowledge, have not been reported previously.

### Predictive ability of risk factors

The RMSE values shown in Tables 3, 4, and 5 describe the ability of the models to predict each outcome variable. A variable associated with a high RMSE means that a substantial part of its variation is not explained by the risk factors included in the model.

For the two flow territory analyses (Tables 3 and 4), the RMSE values vary among the regions. Small noise artefacts remaining in the vessel segmentations probably affect the predictions in the flow territories but not those of the main arteries mean radius, that show lower RMSE values (Table 5), as the main artery masks encompass smaller regions. In the third PLSR model (Table 5), the BA radius prediction resulted in a lower RMSE than all other arteries. This artery is big enough to be accurately segmented in the TOF MRA datasets, while its morphology seems to be well explained by the factors considered in this study. Generally, these findings suggest that there might be other factors, such as imaging and additional environmental and genetic factors, that were not included in the models used in this study and that might explain additional variability.

Considerable differences between the prediction error of corresponding left and right measurements were observed for some of the arteries and flow territories. A potential explanation for these discrepancies could be the imperfect symmetry of the flow territory atlas and the main artery masks. Moreover, it was previously shown that handedness can affect the cerebral blood flow, with higher cerebral blood flow in the right hemisphere for right-handed subjects^27^, which might affect the arteries too. Bullit et al.^11^, who studied the vessel morphology in the left and right MCA territories, among others, also observed discrepancies between the two hemispheric measurements, but did not discuss them.

### Factors affecting artery radius and density

#### ICV and sex

Larger total intracranial volume was associated with an increased artery radius in the flow territories and the main arteries. One potential explanation for this finding is that more brain tissue requires more blood supply so that larger arteries are required. This finding is in line with previous literature reporting a positive association between total cerebral blood flow and ICV^28^. However, as no arterial spin labeling MRI data or similar measurements were available, we can only speculate about the actual association of vascular morphology and cerebral blood flow in the brain tissue. ICV was also negatively correlated with the artery density in the flow territories, which might reflect a reduction of the number of small arteries or their thickness.

Including the intracranial volume variable in the models allows assessing the effects of sex independent of the variability due to intracranial volume differences between males and females. The results showed varying effects of sex depending on the arteries, with larger MCA M1 segments and ICAs for males and a larger ACA A2 segment found in females. Within the flow territories, the MCA and ACA territories showed a significantly larger mean artery radius for males. In line with this finding, Bullitt et al.^11^ and Stefani et al.^29^ also observed sex-related differences of the vessel radius. The former reported a larger average vessel radius for males in four vessel subtrees corresponding to the posterior, left and right middle, and anterior cerebral circulation, which might be comparable to our mean artery radius in the flow territories. The latter studied the main artery diameter using transversal cuts of the arteries around the circle of Willis in adults and found the arteries of the posterior circulation (the PCA and BA) to be larger in males than females, while the present results did not show any sex-related effects in these arteries. Considering the small number of datasets and the use of transversal cuts instead of the mean radius over a larger artery section in this previous study, the results of the present study are likely more robust and reliable. Finally, the artery density showed consistent results in all flow territories with higher density in females compared to males, which is in line with previous findings^11^.

A potential explanation for the observed sex differences are sex steroid hormones, including testosterone, progesterone, and estrogen. For example, it has been shown that estrogen can modulate the cerebral blood flow, decrease vascular tone, and provide vascular protection^30^. The fact that premenopausal females are less prone to strokes also supports the assumption of protective effects of estrogen since estrogen level decrease after menopause^31^. Thus, the observed differences in cerebrovascular morphology between males and females, and especially the higher artery density in women compared to males, could be a result of differences in sex steroid hormones.

#### Age

The BA, PCA, and ACA main artery radii, as well as the artery densities in the associated flow territories significantly decrease with age. Concerning the flow territory artery mean radius, only the PCA region showed a significant mean radius decrease with age. The strong deterioration of the posterior cerebral circulation was also observed by Bullit et al.^11^, who reported the PCA flow territory as being the region with the greatest loss of vessels with aging, although they also found that the PCA enlarges with increased age. However, they did not consider the total intracranial volume in their analysis. The observed decrease of the average radius of most arteries with age is well in line with age-related cerebral blood flow reductions previously observed^10^. Overall, this could also be a result of degeneration and sclerosis of the media layer of the large arteries although this remains speculative.

In contrast to these findings, the ICA is the only artery for which the mean radius was not significantly associated with aging. A previous study investigating the morphological alteration of the ICA with age in 300 adult subjects using carotid contrast-enhanced MRA reported the ICA diameter to increase with age^32^. However, these ICA diameters were measured at the base of the ICA, two centimeters above the carotid artery bifurcation, which might explain the conflicting results as morphological age-related changes of the ICA might vary depending on the measurement location. This was also evident for the MCA measurements, which showed diverging results across the MCA segments. The biological reason for this remains rather speculative and needs further investigation.

#### Obesity

BMI and WHR effects were significant for most of the main artery measurements, where a negative impact on the artery radius was identified. Likewise, the PCA and MCA territories showed a significantly lower artery density with increased WHR and BMI and a decreasing mean artery radius with higher BMI. In line with this finding, Dorrance et al.^33^ reported artery remodelling leading to reduced lumen diameter of the MCAs in diet induced obesity in rats. Marini et al.^34^ associated greater WHR with increased risk of several cerebrovascular diseases, supporting the evidence of a negative impact of obesity on the cerebrovascular structures. Selim et al.^35^ found obesity to be correlated with reduced blood flow velocity in the MCA although no alteration of the MCA diameter was found. The results of the present study support the association of obesity with alterations in the MCA regions.

#### Systolic blood pressure and heart rate

Increased systolic blood pressure was associated with a lower radius in all arteries and flow territories. This factor also showed a significant negative association with the vessel density in all three flow territories. In line with this finding, hypertension was previously reported by several studies as a risk factor for atherosclerosis and vessel rarefaction^36,37^. Iadecola et al.^38^ also identified hypertension as a leading factor responsible for hypertrophic remodeling of the large cerebral arteries, inducing vessel lumen reduction and media thickening, confirming the results of this study as the measurements represent the lumen only. Contrary to this, the heart rate showed varying effects depending on the regions and measurements. More research is needed to investigate these findings in more detail.

#### Smoking duration and alcohol consumption

Overall, smoking duration and alcohol consumption showed contradictory effects on the artery density and radius in the different regions. However, these effects were not significant. To our knowledge, there is no description of the localized effects of these behavioural factors in literature yet. However, general consequences of alcohol and nicotine consumptions have been described. For example, Duzzaro et al.^39^ reported chronic cigarette smoking to be correlated with decreased brain perfusion, while Csordas et al.^40^ observed plaque formation in smokers, eventually leading to atherothrombosis. Evidence of the effects of alcohol consumption on the other hand is not as straight forward. For example, O’Keefe et al.^41^ showed that a light consumption of alcohol reduces cardiovascular risk while greater consumption increases cardiovascular risk. A similar non-linear trend was observed in a study investigating cerebral blood flow, with increased cerebral blood flow in light drinking subjects, and decreased in strong drinking subject^42^. The statistical method used in this study is likely not able to identify such a complex non-linear relationship.

### Limitations

This study used TOF MRA datasets as the basis to quantify the arterial morphology in different flow territories and arteries. The TOF MRA datasets used in this study were acquired without exogenous contrast agent, whereas the blood flow is used as an intrinsic contrast agent to enhance the arteries. Therefore, the artery radii are measurements of the vessel lumen only and the presence of plaque or arterial wall thickening cannot be quantified directly from this imaging sequence. Moreover, the spatial resolution of the image sequence does not allow for imaging of very small vessels, constraining the artery density computation to large and medium sized arteries only.

Another limitation is the lack of additional information about the subjects. In particular, physical exercise has been previously reported to affect the cerebrovascular morphology^43^. However, this information was not available in the dataset used for this study. Within this context, it should also be mentioned that the parameters investigated are a snapshot and do not account for longitudinal changes due to the cross-sectional study design, which might also be one of the reasons for the unexplained variation.

Finally, our results rely on several image pre-processing and feature extraction steps that might introduce some bias in the measurements. In the future, studying the images themselves directly using advanced machine learning approaches would reduce the uncertainty of the results.

## Conclusion

This study investigated the impact of aging and other cardiovascular risk factors on the artery morphology in a large population of healthy adults. We found that several of these risk factors have a significant negative impact on the cerebrovascular morphology. Our results improve the understanding of the cerebrovascular morphological changes associated with cardiovascular risk factors, which is necessary for improved diagnosis and prevention of cerebrovascular and neurological diseases.

## Supplementary Information

Supplementary Information.

## Data Availability

The data that support the findings of this study are available from the Study of Health in Pomerania study upon reasonable request (https://www2.medizin.uni-greifswald.de/cm/fv/ship/).
