# Supplementary material for: Influence of cardiovascular risk-factors on morphological changes of cerebral arteries in healthy adults across the life span
Source: Sci Rep. 2021 Jun 10;11:12236. doi: 10.1038/s41598-021-91669-3 (PMC8192575; doi:10.1038/s41598-021-91669-3)
Supplement: Supplementary file 1 — Supplementary Information. [file 41598_2021_91669_MOESM1_ESM.pdf]

SUPPLEMENTAL MATERIAL

Influence of cardiovascular risk-factors on morphological changes of cerebral arteries in healthy adults across the life span

Pauline Mouches[1,2], Sönke Langner[3], Martin Domin[4], Michael D. Hill[1,2,5,6], Nils D. Forkert[1,2,5,7]

[1] Department of Radiology, University of Calgary, Calgary, Canada

[2] Hotchkiss Brain Institute, University of Calgary, Calgary, Canada

[3] Institute for Diagnostic Radiology and Neuroradiology, University Medicine Rostock, Rostock, Germany

[4] Functional Imaging Unit, Institute for Diagnostic Radiology and Neuroradiology, University Medicine Greifswald, Greifswald, Germany

[5] Department of Clinical Neurosciences, University of Calgary, Calgary, Canada

[6] Department of Community Health Sciences, University of Calgary, Calgary, Canada

[7] Alberta Children's Hospital Research Institute, University of Calgary, Calgary, Canada

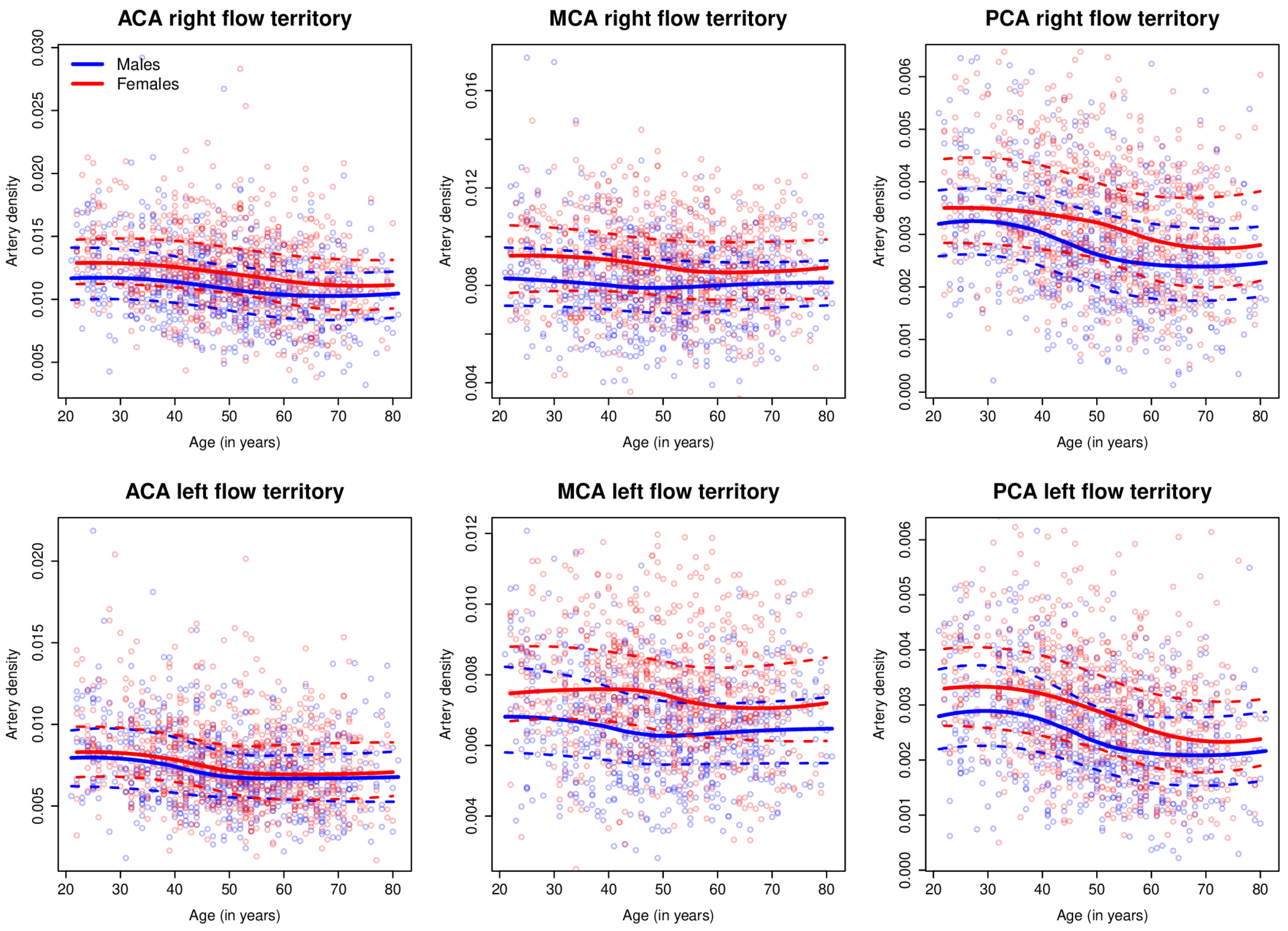

Supplemental Figure 1: Quantile LOESS age- and sex-specific regression plots of the artery density in the left and right anterior cerebral artery (ACA), middle cerebral artery (MCA) and posterior cerebral artery (PCA) flow territories. Solid line: 50th quantile, Dashed lines: 25th and 75th quantiles.

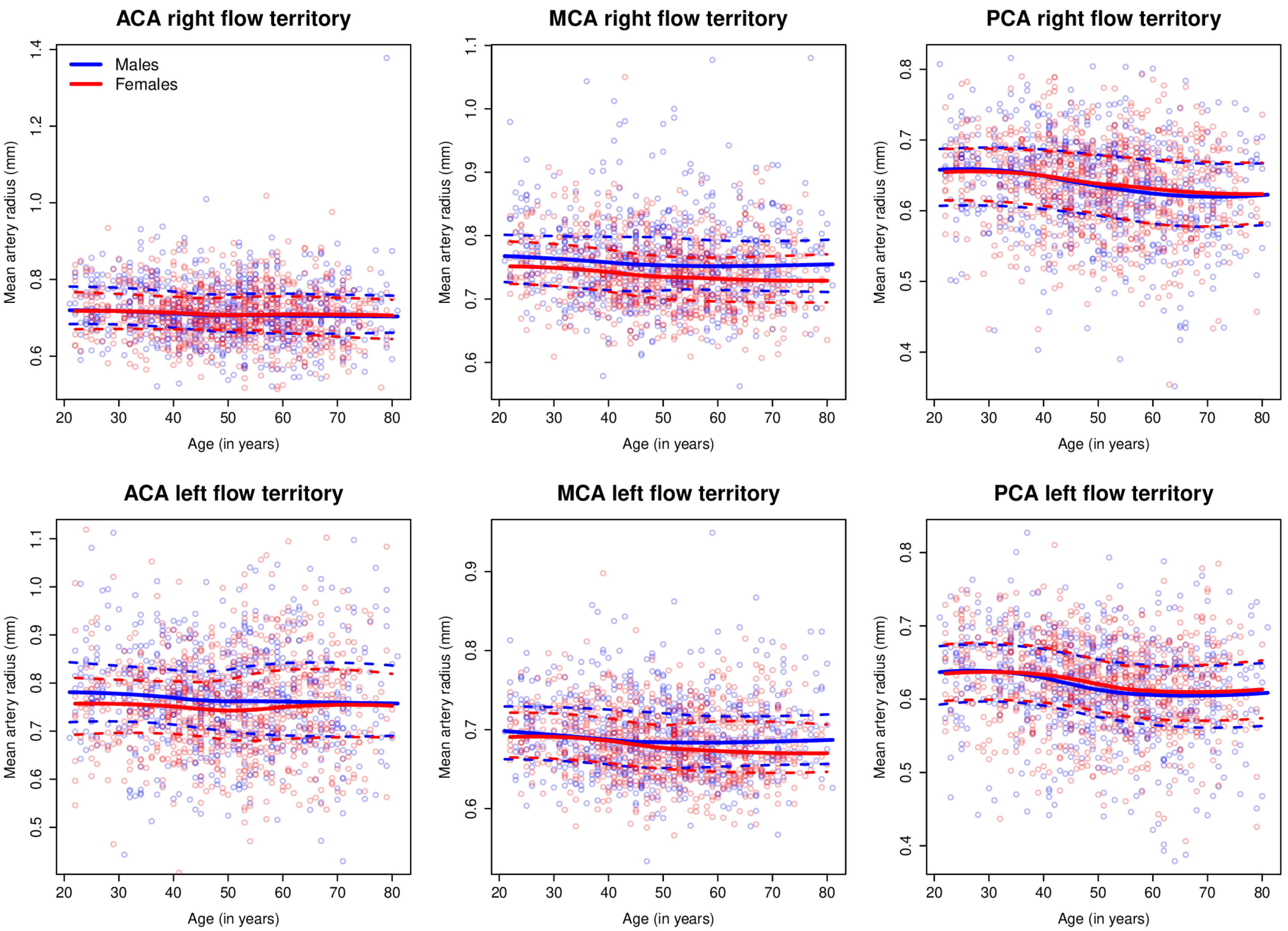

Supplemental Figure 2: Quantile LOESS age- and sex-specific regression plots of the mean artery radius in the left and right anterior cerebral artery (ACA), middle cerebral artery (MCA) and posterior cerebral artery (PCA) flow territories. Solid line: 50th quantile, Dashed line: 25th and 75th quantiles.

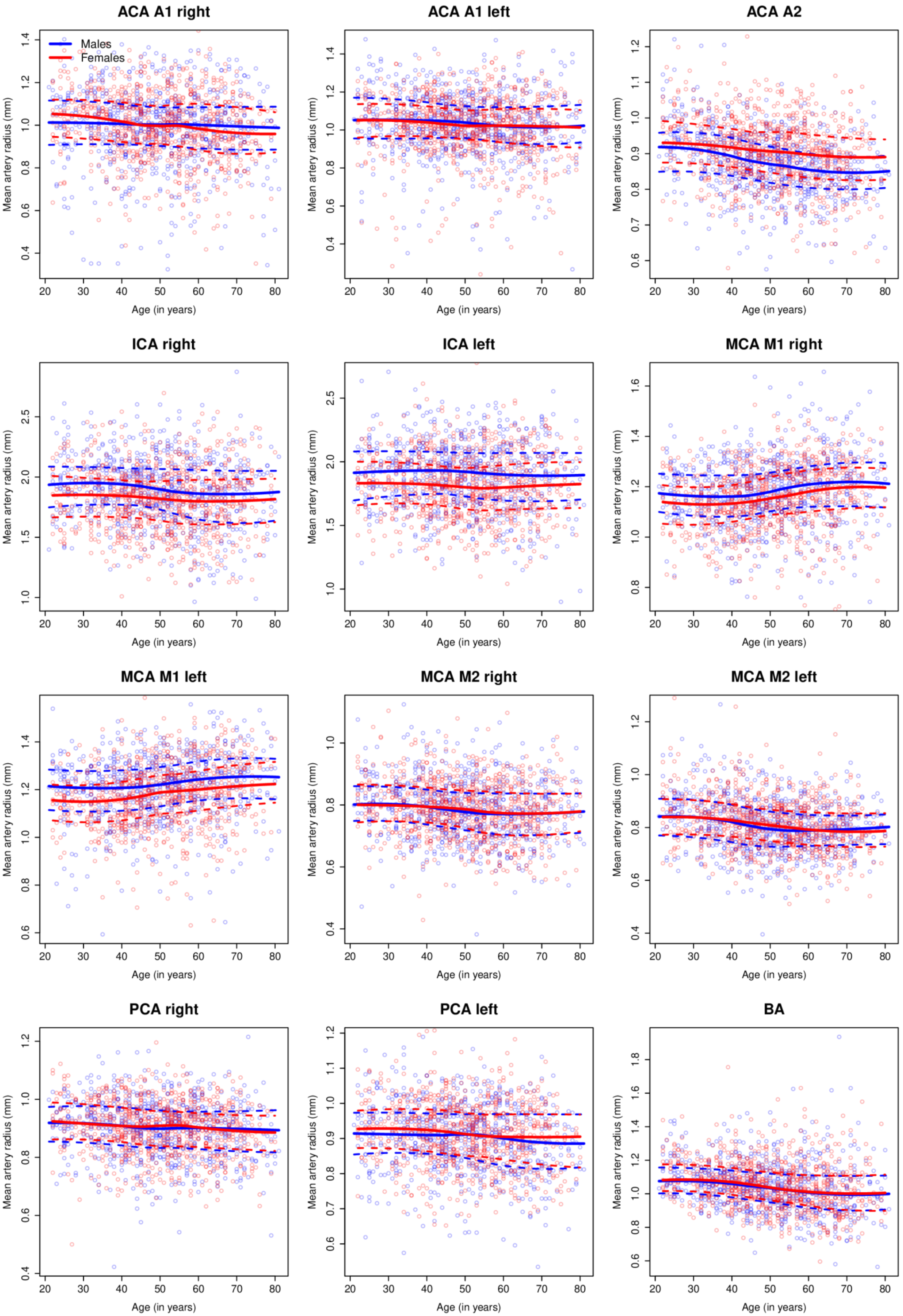
ACA A1 right
Males
Females
ACA A1 left
ACA A2
ICA right
ICA left
MCA M1 right
MCA M1 left
MCA M2 right
MCA M2 left
PCA right
PCA left
BA
Mean artery radius (mm)
Age (in years)

Supplemental Figure 3: Quantile LOESS age- and sex-specific regression plots of the mean artery radius in the left and right anterior cerebral artery (ACA) A1 and A2 segments, internal carotid artery (ICA), middle cerebral artery (MCA) M1 and M2 segments, posterior cerebral artery (PCA), and basilar artery (BA). Solid line: 50th quantile, Dashed line: 25th and 75th quantiles.
